# Tension type headache

**DOI:** 10.1186/1129-2377-14-S1-O2

**Published:** 2013-02-21

**Authors:** Lars Bendtsen

**Affiliations:** 1 Danish Headache Center, Department of Neurology, Glostrup Hostpial, University of Copenhagen, Denmark

## 

Tension-type headache is a common primary headache with high socioeconomic impact. Establishment of an accurate diagnosis is important before initiation of any treatment. Non-drug management is crucial. Information, reassurance and identification of trigger factors may be rewarding. Psychological treatments with scientific evidence for efficacy include relaxation training, EMG biofeedback and cognitive-behavioural therapy. Physical therapy and acupuncture are widely used, but the scientific evidence for efficacy is sparse. Simple analgesics are the mainstays for treatment of episodic TTH. Combination analgesics, triptans, muscle relaxants and opioids should not be used, and it is crucial to avoid frequent and excessive use of simple analgesics to prevent the development of medication-overuse headache. The tricyclic antidepressant amitriptyline is drug of first choice for the prophylactic treatment of chronic TTH, while the antidepressants mirtazapine or venlafaxine are drugs of second choice. Treatment of chronic TTH may be difficult and multidisciplinary treatment strategies are recommended.

